# ​Circulating Cytokines in Myocardial Infarction Are Associated With Coronary Blood Flow

**DOI:** 10.3389/fimmu.2022.837642

**Published:** 2022-02-15

**Authors:** Anna Kalinskaya, Oleg Dukhin, Anna Lebedeva, Elena Maryukhnich, Georgy Rusakovich, Daria Vorobyeva, Alexander Shpektor, Leonid Margolis, Elena Vasilieva

**Affiliations:** ^1^ Laboratory of Atherothrombosis, Cardiology Department, Moscow State University of Medicine and Dentistry, Moscow, Russia; ^2^ Clinical City Hospital named after I.V. Davydovsky, Moscow Department of Healthcare, Moscow, Russia; ^3^ Eunice Kennedy Shriver National Institute of Child Health and Human Development, National Institutes of Health, Bethesda, MD, United States

**Keywords:** myocardial infarction, inflammation, cytokines, clot formation, endothelium

## Abstract

**Background:**

The level of systemic inflammation correlates with the severity of the clinical course of acute myocardial infarction (AMI). It has been shown that circulating cytokines and endothelial dysfunction play an important role in the process of clot formation. The aim of our study was to assess the concentration of various circulating cytokines, endothelial function and blood clotting in AMI patients depending on the blood flow through the infarction-related artery (IRA).

**Methods:**

We included 75 patients with AMI. 58 presented with ST-elevation myocardial infarction (STEMI) and 17 had non-ST-elevation myocardial infarction (non-STEMI). A flow-mediated dilation test (FMD test), thrombodynamics and rotational thromboelastometry as well as assessment of 14 serum cytokines using xMAP technology were performed.

**Findings:**

Non-STEMI-patients were characterized by higher levels of MDC, MIP-1β, TNF-α. Moreover, we observed that patients with impaired blood flow through the IRA (TIMI flow 0-1) had higher average and initial clot growth rates, earlier onset of spontaneous clots, C-reactive protein (CRP) and IL-10 compared to patients with preserved blood flow through the IRA (TIMI flow 2-3). Patients with TIMI 2-3 blood flow had higher level of IP-10. IL-10 correlated with CRP and pro-inflammatory cytokines levels, initial clot growth rate and clot lysis time in TIMI 0-1 patients. All these differences were statistically significant.

**Interpretation:**

We demonstrated that concentrations of the inflammatory cytokines correlate not only with the form of myocardial infarction (STEMI or non-STEMI), but also with the blood flow through the infarct-related artery. Inflammatory response, functional state of endothelium, and clot formation are closely linked with each other. A combination of these parameters affects the patency of the infarct-related artery.

## Introduction

The cornerstone in the pathogenesis of acute myocardial infarction (AMI) is the interaction between the endothelium, hemostasis and the chronic inflammation that accompanies atherosclerosis (1, 2).

Currently, AMI is divided into ST-elevation myocardial infarction (STEMI) and non-ST-elevation myocardial infarction (non-STEMI) according to electrocardiogram at presentation (3). These types of AMI differ in the blood flow through the infarct-related artery (IRA): while the majority of patients with STEMI demonstrate occlusion of IRA on coronary angiography, the development of IRA occlusion in patients with non-STEMI is uncommon. Nevertheless, approximately 4-24% of STEMI cases are characterized by the development of spontaneous reperfusion (SR) of the IRA (4), while up to 25% of non-STEMI cases may be accompanied by occlusion (5).

Despite the fact that certain determinants of the patency of the IRA are well-known (younger age, the absence of significant concomitant diseases, a lower contents of lipoprotein (a), total cholesterol, homocysteine, etc.), the exact mechanism of blood flow preservation through the IRA remains unclear (6–10).

Earlier, we demonstrated that the functional state of endothelium as assessed with flow-mediated dilation test (FMD test) correlates with the patency of IRA (11). The higher activity of endogenous fibrinolysis, which is closely interconnected with the functional state of endothelium, is the most probable cause of SR (12–15). Also, several small studies demonstrated higher platelet reactivity in patients without SR of the IRA (16, 17).

The existing data show the general dependence of the occlusion of the IRA and long-term prognosis on the inflammatory status of AMI-patients. While various cytokines play an important role in inflammation, it is mostly evaluated from measurement of high sensitive C-reactive protein (hs-CRP) together with leukocytes counts. Little is known about the possible role of cytokines in the state of blood flow through the IRA (18).

Here, we attempted to fill in this gap. The aim of the current study was to investigate the links between the functional state of the endothelium, the cytokine spectrum, and critical parameters of thrombus formation in AMI-patients with differences in coronary blood flow through the IRA.

## Methods

### Subjects

The study was performed at the Moscow City Clinical Hospital named after I.V. Davydovsky between January 2017 and October 2021. The study protocol was developed in accordance with the principles of the Helsinki Declaration and was approved by the Interuniversity Committee of Ethics. All participants signed an informed written consent.

We included 75 AMI patients in our study: 58 presented with STEMI, while 17 presented with non-STEMI. All patients underwent selective coronary angiography with subsequent primary percutaneous coronary intervention (PCI) (STEMI-patients directly upon admission, non-STEMI patients during the first 24 hours of hospitalization). Patency of the IRA was determined on TIMI Grade flow classification (Appleby et al., 2000). Of the patients, 45 had an impaired blood flow through the IRA (TIMI 0-1) and 30 were characterized by preserved blood flow (TIMI 2-3).

Inclusion criteria: STEMI or non-STEMI patients; onset of symptoms during the 24 hours before admission; informed written consent.

Exclusion criteria: Age over 90 or under 18; more than 24 hours from the onset of symptoms, thrombolytic therapy, signs of cardiogenic shock, acute and chronic infectious diseases and inflammatory processes, severe anemia or ongoing bleeding, pregnancy, known oncological process, anticoagulant therapy.

In addition to the standard clinical examination, on admission, all patients underwent: a flow-mediated dilation test (FMD test) to evaluate endothelial function, thrombodynamics and rotational thromboelastometry (in NATEM mode) to evaluate plasma coagulation, thrombodynamics in fibrinolysis mode to evaluate endogenous fibrinolysis, and xMAP technology to measure concentration of 40 cytokines.

### Blood Sampling

A 21-gauge needle was used with minimal stasis in order to avoid iatrogenic induction of platelet aggregation, and peripheral venous blood was drawn in the amount of 4.5 ml into a tube containing 0.105 M buffered sodium citrate anticoagulant. The whole blood was used for rotational thromboelastometry. Platelet-poor plasma was used for the thrombodynamics study. Blood was centrifuged at 1,600 g for 15 min to obtain platelet-poor plasma. Subsequently, the platelet-poor plasma was transferred to a new centrifugation tube and centrifuged at 10,000 g for 15 min to obtain platelet-free plasma.

### Rotational Thromboelastometry

We carried out the study using ROTEM (Roche). The study was performed in NATEM mode. The following indicators were used: clotting time (CT, sec) and thrombus amplitude at different time sections of the study (A10, A20, mm).

### Thrombodynamics

The study was performed on a T-2 Thrombodynamics Analyzer according to a standard technique (19). The following parameters of clot growth were used: clot growth rate (V, μm/min), initial clot growth rate (Vi, μm/min), and spontaneous clot formation time (Tsp, min). A standard activator with urokinase was added to induce thrombus lysis. The lysis was characterized by the time of lysis onset (LOT, min), clot lysis time (CLT, min), and the rate of lysis progression (LP, %/min).

### FMD Test

To assess endothelial function, all patients underwent an FMD test according to the standard method (20). The results of FMD-test were expressed in %.

### Cytokine Quantification

40 cytokines in serum were measured with a commercial kit, MILLIPLEX MAP Human Cytokine/Chemokine Magnetic Bead Panel (Merсk Millipore). The cytokine panel included interleukin-1α (IL-1α), IL-1β, IL-1RA (IL-1 receptor antagonist), IL-2, IL-3, IL-4, IL-5, IL-6, IL-7, IL-8, IL-9, IL-10, IL-12 (p40), IL-12 (p70), IL-13, IL-15, IL-17A, fractalkine (CX3CL1), growth-regulated alpha (GRO-α or CXCL1), interferon-γ-induced protein-10 (IP-10 or CXCL10), monocyte chemoattractant protein-1 (MCP-1 or CCL2), MCP-3 (CCL7), macrophage inflammatory protein-1α (MIP-1α or CCL3), MIP-1β (CCL4), eotaxin (CCL11), macrophage-derived chemokine (MDC or CCL22), soluble CD40-ligand (sCD40L), epidermal growth factor (EGF), fibroblast growth factor-2 (FGF-2), Fms-like tyrosine kinase 3 ligand (Flt-3L), vascular endothelial growth factor (VEGF), granulocyte colony-stimulating factor (G-CSF), granulocyte-macrophage colony-stimulating factor (GM-CSF), platelet-derived growth factor-AA (PDGF-AA), PDGF-AB/BB, transforming growth factor-α (TGF-α), interferon-α2 (IFN-α2), IFN-γ, tumor necrosis factor-α (TNF-α), and TNF-β. All cytokines were measured in pg/ml. Serum was diluted 4 times in assay buffer to reduce the matrix effect and added in a volume of 50 µl to each well. The standard curve was built up from 8 standard dilutions in triplicates, with the 1^st^-3^rd^ standard dilutions with dilution factor 5 and the and 4^th^-8^th^ dilutions with dilution factor 4. We used serum matrix diluted in assay buffer to mimic the matrix effect on the standard curve, controls, and blank wells. Standards and controls (25 µl) were diluted with 25 µl of serum matrix. We added 15 µl of 40-plex magnetic beads to each well and incubated for 18 h at 4°C and then for 30 min at 25°C. Beads were washed twice with automatic magnetic washer (Biotech ELx405) and incubated with detection antibodies for 1 h at 25°C. Antibodies were diluted with wash buffer in 1,93 times and added in the amount of 25 µl per well. After incubation, we added 15 µl of Streptavidin-PE solution to each well and incubated the final solution for 30 min at 25°C. Then, beads were washed twice, resuspended in the sheath fluid, and analyzed by means of the Luminex 200 system. For the analysis we collected 50 beads per region. Wells with fewer than 20 beads per region were excluded from analysis. During the analysis, we used 5PL fit for the standard curve.

During explanatory analysis we considered 26 from 40 initially measured cytokines in 40% of cases to be under the detection limit, and we decided to exclude them from analysis. The following cytokines were available for the final analysis: EGF, Eotaxin, GRO-α, IL-10, IL-8, IP-10, MCP-1, MDC, MIP-1β, PDGF-AA, PDGF-AB/BB, sCD40L, TGF-α, and TNF-α.

### Statistical Analysis

Statistical analysis was performed with R (4.0.5). The expression values obtained in the present study were in most cases not normally distributed, according to the Shapiro-Wilk test. For comparison of several groups, we used the Mann-Whitney rank test with continuity correction. For the analysis of categorical parameters, we used a two-tailed Fisher’s exact test with 2x2 frequency tables. In order to overcome errors from multiple comparisons we performed a Benjamini-Hochberg FDR-correction with calculation of critical values for each comparison matched with corresponding p-values; we calculated adjusted p-values and compared them with a critical value of 0.05, if not stated otherwise. For calculation of Spearman’s coefficient, we used a minimum 9 pairs of observations and a threshold of *p*.adjusted < 0.2 to keep the positive false discovery rate below 20%. 26% of the data was missing at random values in different parameters that did not result in sample bias by definition, so the pairwise deletion approach was used. The available data sets are sufficient to demonstrate significant differences in the cytokine levels using Mann-Whitney rank test, sig. level = 0.05, power = 0.8, and effect size = 0.8 which corresponds to ‘large’ effect size (21).

## Results

### Comparison of STEMI and Non-STEMI Patients

#### Clinical Data

Compared with STEMI-patients, patients with non-STEMI were characterized by a significantly more frequent history of myocardial infarction (40% vs. 2.4%) and PCI (44% vs. 2.5%), and more often presented with multivessel disease (66.7% vs. 24.5%) (**Table 1**).

**Table 1:** Comparison of clinical data of patients in different groups, two types of stratification (STEMI vs. Non-STEMI; TIMI 0-1 vs. TIMI 2-3).

n	STEMI	Non-STEMI	*p*.adjusted
	58	17	-
Age, mean ± sd, years	60.2± 10.5	63.2± 10.0	0.27
Sex (male), %	81%	82,4%	1
Diabetes, %	12.7%	20%	0.61
Arterial hypertension, %	85.2%	93.3%	0.78
History of MI, %	2.3%	40%	<0.01
Smoking, %	50.9%	33.3%	0.45
History of PCI, %	2.5%	44.4%	<0.01
Multivessel disease, %	24.5%	66.7%	0.01
	TIMI 0-1	TIMI 2-3	*p*.adjusted
n	45	30	-
Age, mean ± sd, years	59.2 ± 10.3	63.5 ± 10.1	0.09
Sex (male), %	77.8%	86.7%	0.45
Diabetes, %	15.9%	11.5%	0.73
Arterial hypertension, %	81.8%	96%	0.2
History of MI, %	0%	29.1%	<0.01
Smoking, %	54.5%	34.6%	0.2
History of PCI, %	3%	25%	0.08
Multivessel disease, %	23.8%	50%	0.08

p-value < 0.05 is statistically significant.

#### FMD-Test

There was no significant difference in FMD-test values between STEMI and non-STEMI patients.

#### Immune Mediators

**Figure 1:**
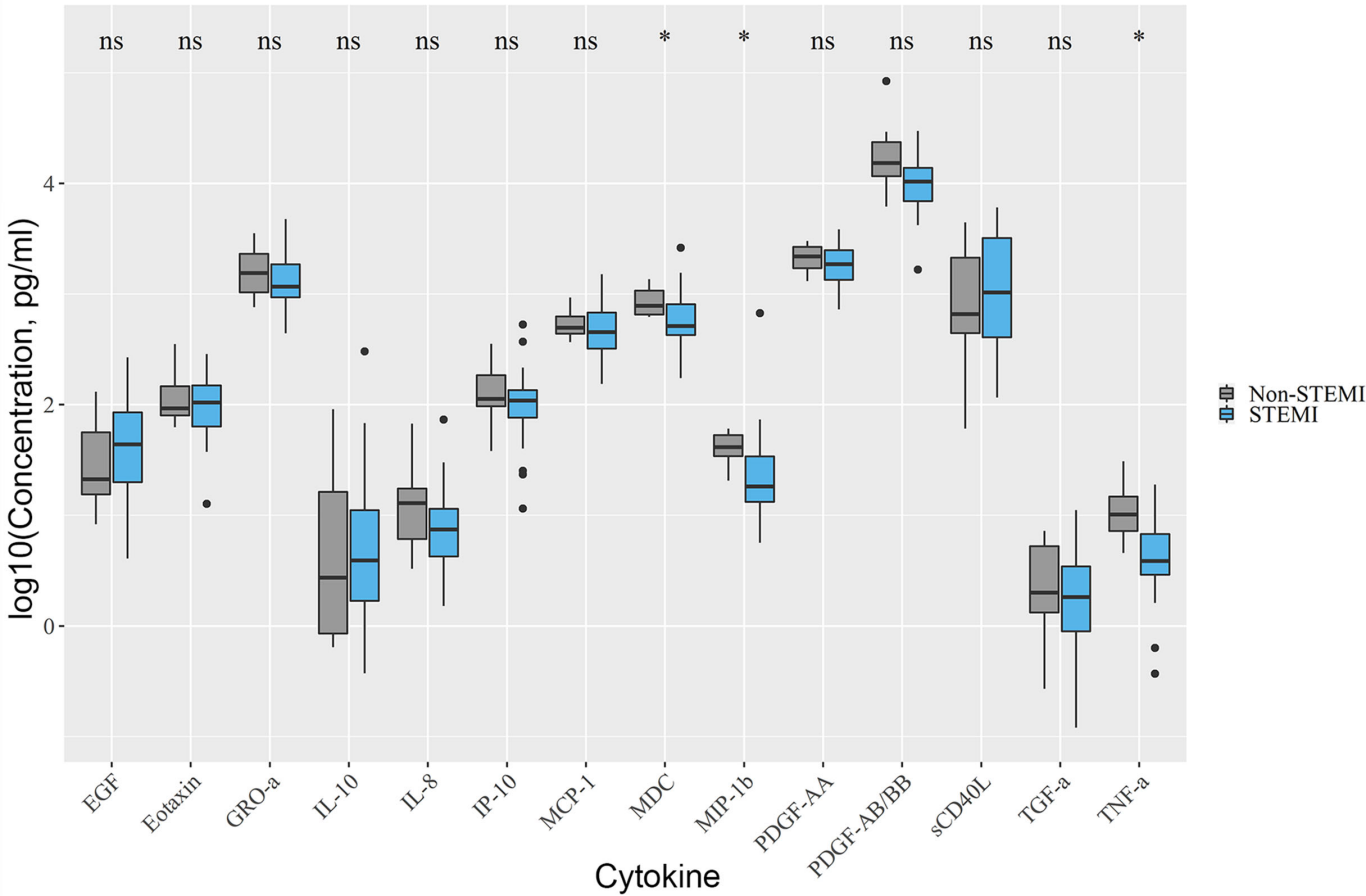
Differences in cytokine levels between STEMI and non-STEMI patients. Non-STEMI patients had elevated levels of MDC, MIP-1β, TNF-α. Asterix indicates p-adjusted < 0.05. ns, not significant.

Non-STEMI patients had higher levels of MDC (779.13 [654.5; 1070.7]) vs. 513.63 [426.7; 807.95], *p*.adj < 0.05 in STEMI), MIP-1β (41.1 [34.0; 53.4]) vs. 18.1 [13.3; 34.1], *p*.adj < 0.05 in STEMI), TNF-α (10.2 [7.2; 14.8]) vs. 3.84 [2.9; 6.77], *p*.adj < 0.05 in STEMI) compared with STEMI-patients (**Figure 1**).

#### Clot Formation and Endogenous Fibrinolysis

There were no differences between STEMI and non-STEMI patients in parameters of clot formation and endogenous fibrinolysis. We observed a positive moderate strength correlation between TNF-α with LOT (r=0.4, *p* < 0.05, *p*.adj < 0.2) and CLT (r=0.5, *p* < 0.05, *p*.adj < 0.2).

### Comparison of AMI-Patients With Different Blood Flow Through the IRA

#### Clinical Data

Patients with preserved blood flow through the IRA had a history of myocardial infarction more frequently (29.1% vs. 0% in TIMI 0-1) (**Table 1**).

#### FMD Test

Patients with TIMI 2-3 blood flow were characterized by higher values of FMD test results (5.71 [4.3; 9.1] vs. 4.28 [2.5; 5.7] in TIMI 0-1, *p*.adj < 0.05).

#### Immune Mediators

**Figure 2:**
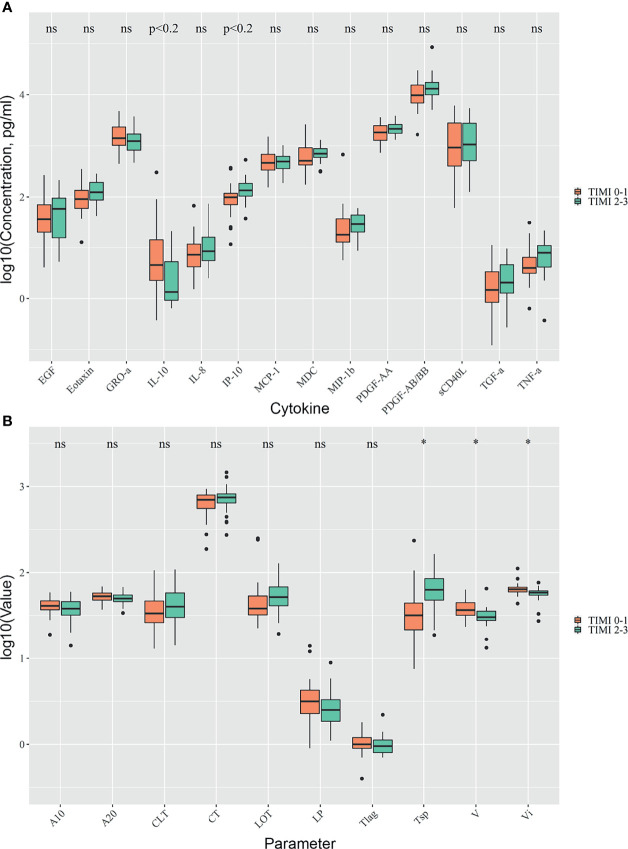
Comparison of TIMI 0-1 and TIMI 2-3 groups. Differences in cytokine concentrations **(A)**, clot formation and endogenous fibrinolysis **(B)**. Asterix indicates p-adjusted < 0.05, ns, not significant.

Patients with TIMI 2-3 blood flow were characterized by a higher level of IP-10 (133.58 [103.7; 184] vs. 97.8 [70.7; 118.3] in TIMI 0-1, *p* < 0.05, *p*.adj < 0.2) while patients with TIMI 0-1 blood flow had higher levels of IL-10 (4.5 [2.3; 14.1] vs. 1.3 [0.9; 5.3] in TIMI 2-3, *p*< 0.05, *p*.adj < 0.2) (**Figure 2A**). Hs-CRP levels were higher in patients with TIMI 0-1 blood flow (5.4 [2.8; 18.2] vs. 0.1 [0.1; 0.95] in TIMI 2-3, *p* < 0.05). We analyzed the predictive value of studied cytokines and found IP-10 to be the most effective in separating between subject groups with TIMI 0-1 and 2-3, with ROC AUC = 0.72, and optimal sensitivity/specificity at 0.64/0.30, respectively (**Figure S1**).

#### Clot Formation and Endogenous Fibrinolysis

Patients with TIMI 0-1 blood flow had higher average and initial clot growth rates (36.6 [31.9; 44.8] vs. 30.4 [27.9; 35.7] in TIMI 2-3, and 64.05 [59.8; 67.8] vs. 58.6 [54.6; 61.3] in TIMI 2-3, *p*.adj < 0.05) and earlier onset of spontaneous clots (31.8 [21.6; 44.4] vs. 63.6 [48; 85.4] in TIMI 2-3, *p*.adj < 0.05) (**Figure 2B**).

#### Correlations Between Immune Mediators and Parameters of Haemostasis

**Figure 3:**
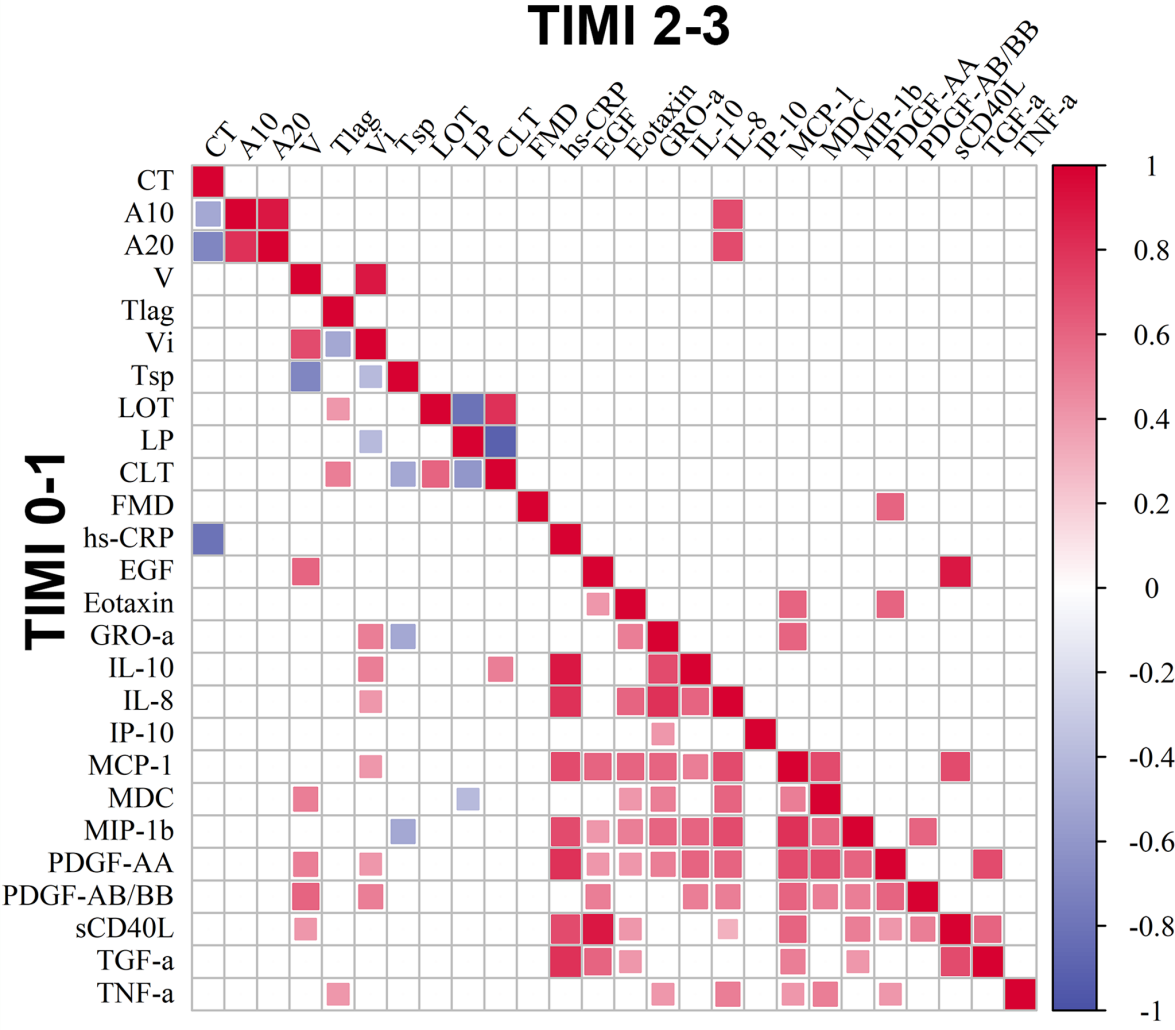
Correlation matrix of circulating markers of inflammation, clot formation and endogenous fibrinolysis and FMD. Included are 14 cytokines, hs-CRP, FMD and clot formation and endogenous fibrinolysis parameters in groups of patients with TIMI 0-1 and TIMI 2-3 blood flow, *p* < 0.05, *p*.adj < 0.2.

We observed significant positive correlations between IL-10 with the levels of hs-CRP and pro-inflammatory chemokines such as IL-8, GRO-α, MIP-1β, MCP-1, as well as with the Vi and CLT in TIMI 0-1 patients (**Figure 3**).

We performed an additional analysis of the influence of diabetes mellitus on the observed correlations by exclusion these patients from the cohort. The correlations described above were mostly preserved but tended to have lower correlation coefficients (**Figure S2**).

## Discussion

It is firmly established that chronic inflammation is the driving force of atherosclerosis (2, 22). There is a large body of evidence concerning the active involvement of leukocytes in the formation of atherosclerotic plaque (23, 24). Also, leukocytes promote proliferation of smooth muscle cells and their phenotypic switch, facilitating further plaque growth (25). The immune system plays a key role in the destabilization of atherosclerotic plaque, the most common cause of AMI, primarily through the massive release of matrix metalloproteinases by macrophages and impaired collagen synthesis (26–28). All the above processes are finely regulated by inflammatory and anti-inflammatory cytokines.

The level of systemic inflammation and prothrombotic state correlates with the severity of AMI (29). The positive effect of inhibition of systemic inflammation with statins and PCSK9 inhibitors both on reducing markers of systemic inflammation (CRP, etc.) and on major adverse cardiovascular events has been previously demonstrated in a number of clinical studies (30–34). Also, in a randomized double-blind clinical trial, CANTOS, a significant reduction in the number of adverse cardiovascular diseases was demonstrated under the influence of the specific IL-1β blocker, canakinumab (35). Considering this, the study of the effect of various cytokines both on the course of AMI and on the state of blood flow in the IRA facilitate understanding the mechanisms of relation of inflammation to AMI.

In pursuing this aim, we choose the panel of cytokines that are known to be associated with inflammation and haemostasis (36–40). Several cytokines in our panel such as IL-10, TNF-α, MCP-1, GRO-α, IP-10 and IL-8 were shown to be associated with the course and with the outcome of AMI (41–43). IL-10, TGF-α, IP-10, MIP-1β, IL-8, and Eotaxin were found to be predictors of AMI in the study by Hoogeveen et al. (44). Furthermore, as the goal of our study was also to investigate the influence of immune mechanisms on the prothrombotic state in AMI, we included in our study several cytokines that may not be directly connected to thrombosis but can be involved in the network and reflect the status of different cell types during AMI.

In the first part of our observational study we evaluated the levels of 14 circulatory cytokines in patients with STEMI and non-STEMI with differences in blood flow through the coronary artery and found significant correlations between these parameters. Non-STEMI patients in our study, as well as in the published data (45), were characterized by a longer history of coronary artery disease, repeated revascularization, and of multivascular involvement. In our study patients with non-STEMI demonstrated significantly higher levels of TNF-α, MDC and MIP-1β compared with STEMI patients.

These cytokines were reported to be directly connected to atherosclerosis development and progression. In particular, TNF-α is a pleiotropic cytokine that is involved in atherothrombosis in several pathways: TNF-α increases the expression of adhesion molecules by endothelium and as well enhances leukocyte adhesion and rolling (46), and it is involved in the implementation of endothelial dysfunction *via* increase of reactive oxygen species production and impairment of NO-mediated vasodilation (47–50). Finally, there is evidence of increased platelet aggregation upon TNF-α stimulation (51–54). MIP-1β was shown to be released by macrophages and smooth muscle cells in atherosclerotic plaques. Atherosclerotic patients had elevated levels of this circulating chemokine (55–57). It has been shown that TNF-α induces MIP-1β expression in THP-1 cells (58). Therefore, a possible mechanism of synergistic action of TNF-α and MIP-1β in AMI patients can be TNF-α-mediated induction of MIP-1β expression in monocytes and increased adhesion of leukocytes to endothelium pre-activated with TNF-α. MDC is predominantly secreted by macrophages and dendritic cells and is involved in migration of monocytes. MDC expression has been demonstrated in human atherosclerotic plaques and associated with M2 macrophages (59). One of the possible actions of MDC in cardiovascular diseases can be mediated by platelet activation as measured by platelet aggregation and calcium flux (60).

The course of AMI and its long-term prognosis depend on the baseline blood flow through the IRA. TIMI flow grade is commonly used to classify blood flow through the IRA (61). In the second part of our study, we investigated the link between cytokine spectrum, haemostasis, endothelium state and blood flow in AMI patients. We used the division of patients into a group with TIMI 0-1 blood flow (which indicates an almost complete absence of distal blood flow through the IRA) and a group with TIMI 2-3 blood flow, characterized by partially or fully preserved blood flow.

First, in these groups we analyzed blood clotting and endogenous fibrinolysis due to IRA blood flow dependence on clot formation and stability. Patients with impaired blood flow (TIMI 0-1) were characterized by more intensive clot formation compared with patients with preserved blood flow (TIMI 2-3). Our results are in agreement with the data on enhanced blood clotting in patients with impaired blood flow through the IRA (16, 17).

Functional state of endothelium and circulating cytokines have a direct influence on parameters of haemostasis. Earlier, it was shown that the FMD test can be a predictive factor of IRA patency and long-term prognosis in patients with AMI (11). In the present study we confirmed these results.

Furthermore, we compared levels of circulating cytokines in TIMI 0-1 and TIMI 2-3 groups of patients and analyzed their correlations with other markers of inflammation, specifically with parameters of coagulation and fibrinolysis. We found that the TIMI 0-1 and TIMI 2-3 groups had different patterns of correlations and most of the correlations were detected in the TIMI 0-1 group, probably because of massive intracoronary thrombosis in patients in this group.

In our study patients with impaired coronary blood flow (TIMI 0-1) had lower levels of IP-10 and higher levels of IL-10, compared with TIMI 2-3 patients. Moreover, IP-10 was found to be the most effective among other analyzed cytokines in separating subject groups with TIMI 0-1 and 2-3 blood flow. The predictive value and clinical implication of this result should be investigated in further prospective studies.

These results can be explained in the context of these cytokine functions. IP-10 is a chemokine belonging to the CXC chemokine family. It is secreted by cells of many types including smooth muscle cells, endothelial cells, and macrophages in atherosclerotic plaques; it acts as a chemoattractant predominantly for lymphocytes (62, 63), and can promote proliferation and migration of smooth muscle cells (38). Several studies have demonstrated upregulation of IP-10 during AMI, and it’s reverse correlation with the infarct size (64, 65). In our study, we have shown that IP-10 is elevated in patients with AMI with TIMI 2-3 coronary blood flow, compared with TIMI 0-1 patients. The patency of IRA can be associated with elevated levels of IP-10 and thus, with smaller infarct size in this group. IL-10 is an anti-inflammatory cytokine that can be released by various immune cells in response to pro-inflammatory cytokines and inhibits their action (66). The protective properties of this cytokine in relation to the development of atherosclerosis and the size of the zone of myocardial infarction were shown in several previous studies (67–70).

In our study, elevated levels of IL-10 can be a response to enhanced tissue injury and inflammation in TIMI 0-1 patients. This is confirmed by IL-10 correlation with the levels of hs-CRP and pro-inflammatory chemokines such as IL-8, GRO-α, MIP-1β, and MCP-1 in TIMI 0-1 patients (71). Elevated IL-10 levels can be not only a consequence of impaired coronary blood flow in TIMI 0-1 patients but can also be connected to the coagulation cascade. Despite the fact that several studies demonstrated an inhibitory effect of IL-10 on coagulation, we found a positive correlation of IL-10 with the initial clot growth rate (Vi) in patients with TIMI 0-1 (37). This correlation can be related to the association of coagulation with the pro-inflammatory response, where IL-10 can be induced by acute inflammation and be a bystander to increased coagulation. This seems to be confirmed by the correlation of IL-10 with pro-inflammatory chemokines such as GRO-α, IL-8, and MCP -1 and also by correlation of these chemokines with Vi (36). On the other hand, IL-10 can be involved in stabilization of the thrombus. Previously, it was shown that during endotoxemia, IL-10 inhibits not only coagulation, but also fibrinolysis (37). Alshehri et al. (72) showed that FXIII-A antigen was up-regulated in human monocytes in response to stimulation by IL-10, and treatment of monocytes with IL-10 stabilized FXIII-depleted thrombi from fibrinolytic degradation. On the basis of these facts taken together, it can be proposed that in AMI IL-10 not only inhibits pro-inflammatory stimuli leading to resolution of inflammation, but also stabilizes thrombus. In our study, this is evidenced by positive correlation of IL-10 with clot lysis time (CLT) in TIMI 0-1 patients. Due to the possible influence of metabolic disorders on the inflammatory status in AMI-patients, we performed an additional analysis excluding patients with diabetes mellitus. The most important correlations were preserved, but as expected, tended to have a lower correlation coefficients, which could be explained not only by the influence of diabetes mellitus, but by the smaller group size as well.

Thus, our findings demonstrated that the circulating cytokines may play an important role in the course of AMI by influencing endothelial function, clot formation, and fibrinolysis. Further research will determine the possible impact of cytokine spectrum on AMI treatment strategy.

## Study Limitations

The main limitation of our study was the relatively small number of patients included and available values, which led to the necessity of raising the threshold of adjusted *p*-value in some cases to reach the significant differences. Because 26 of the 40 initially measured cytokines in 40% of cases were under the detection limit, they were excluded from the final analysis. Nevertheless, these cytokines may also play an important role in AMI and should be analyzed in a larger cohort of patients.

## Conclusion

We demonstrated that inflammatory cytokines correlate not only with the form of myocardial infarction (STEMI or non-STEMI), but also with the blood flow through the infarct-related artery. Inflammatory response, functional state of endothelium, and clot formation are closely linked with each other. A combination of these parameters affects the patency of the infarct-related artery.

## Data Availability Statement

The raw data supporting the conclusions of this article will be made available by the authors, without undue reservation.

## Ethics Statement

The studies involving human participants were reviewed and approved by Davydovsky Moscow City Clinical Hospital local ethics committee. The patients/participants provided their written informed consent to participate in this study.

## Author Contributions

AK, OD, AL, DV, EM, LM, EV designed the study, contributed to the literature search, data collection, data analysis, data interpretation and writing of the manuscript. GR contributed to data analysis, data interpretation and writing of the manuscript. AS, LM, EV critically reviewed the manuscript. All authors contributed to the article and approved the submitted version.

## Funding

The reported study was funded by RFBR, project number 20-315-70047. The work of LM was supported by the NICHD Intramural Program.

## Conflict of Interest

The authors declare that the research was conducted in the absence of any commercial or financial relationships that could be construed as a potential conflict of interest.

## Publisher’s Note

All claims expressed in this article are solely those of the authors and do not necessarily represent those of their affiliated organizations, or those of the publisher, the editors and the reviewers. Any product that may be evaluated in this article, or claim that may be made by its manufacturer, is not guaranteed or endorsed by the publisher.
