# Supplementary material for: ​Circulating Cytokines in Myocardial Infarction Are Associated With Coronary Blood Flow
Source: Front Immunol. 2022 Feb 15;13:837642. doi: 10.3389/fimmu.2022.837642 (PMC8886043; doi:10.3389/fimmu.2022.837642)
Supplement: Supplementary file 1 [file DataSheet_1.pdf]

Supplementary figures

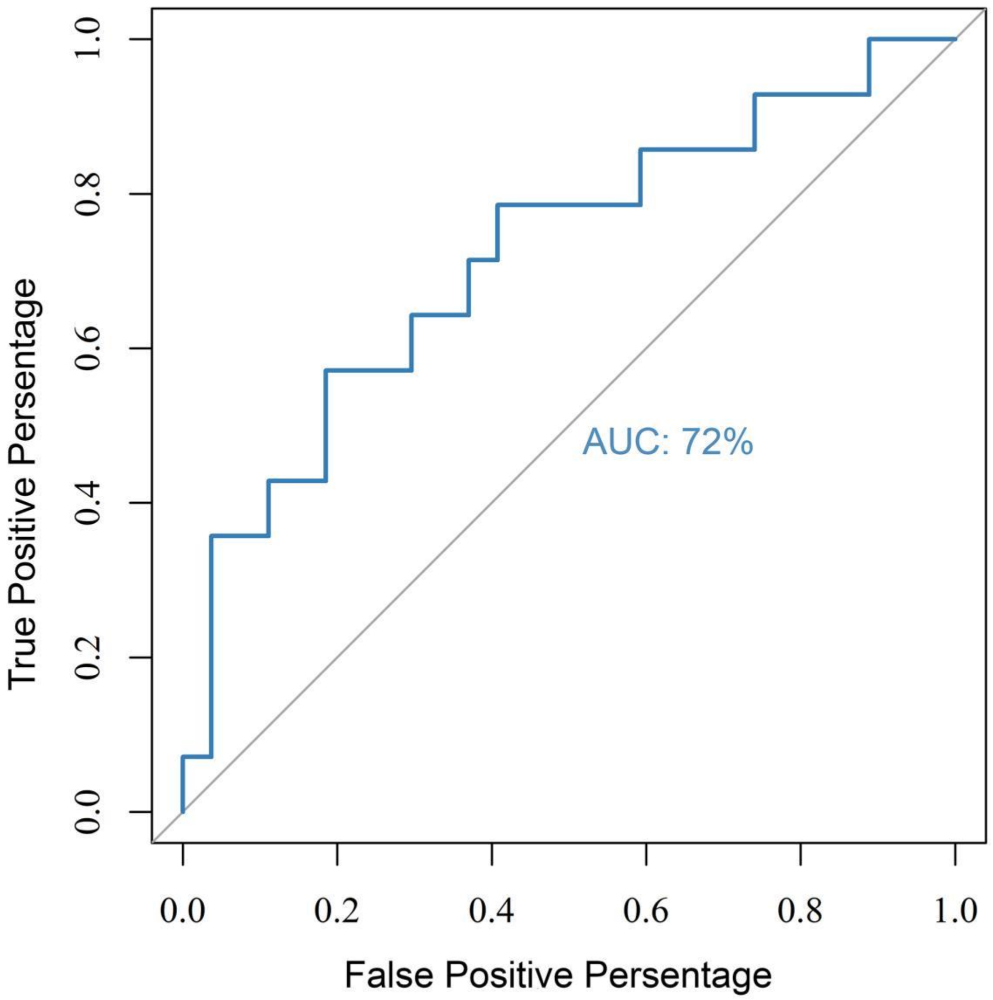

Figure S1. ROC curve for binary classifier separating patients into groups TIMI 0-1 or TIMI 2-3 using IP-10 as a single input parameter.

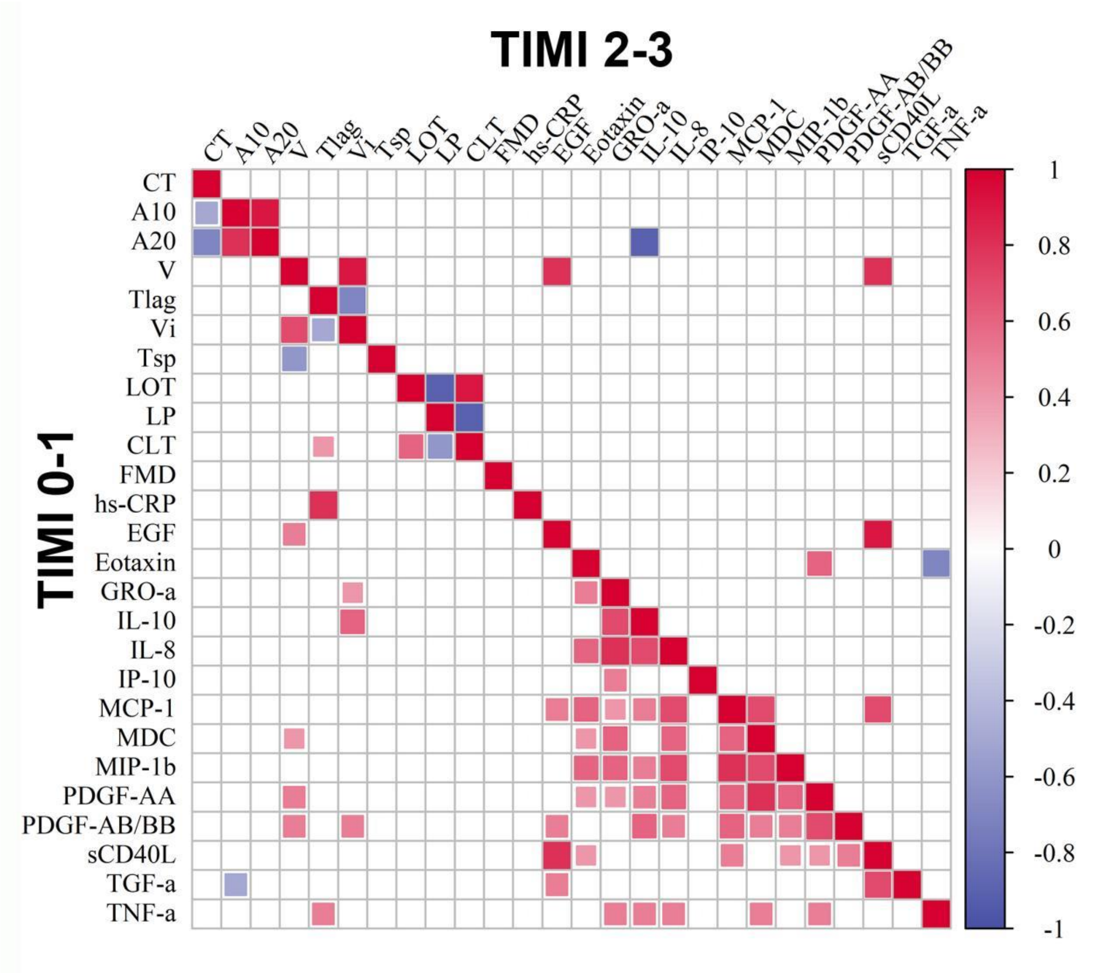

Figure S2. Correlation matrix of circulating markers of inflammation, clot formation and endogenous fibrinolysis and FMD for patients without diabetes. Included are 14 cytokines, hs-CRP, FMD and clot formation and endogenous fibrinolysis parameters in groups of patients with TIMI 0-1 and TIMI 2-3 blood flow, $p < 0.05$, p.adj < 0.2.
